# Disparities in the impact of drought on agriculture across countries

**DOI:** 10.1038/s41598-025-94166-z

**Published:** 2025-04-18

**Authors:** Hayden Freedman, Amir AghaKouchak, Angela J. Rigden, André van der Hoek, Bill Tomlinson

**Affiliations:** 1 Department of Informatics, University of California Irvine, Irvine, 92697 CA USA; 2 Department of Earth System Science, University of California Irvine, Irvine, 92697 CA USA

**Keywords:** Climate-change impacts, Hydrology

## Abstract

Over the last several decades, droughts driven by climate change have damaged agricultural production as the planet warms. It is crucial for the future of the global food supply to develop effective adaptation strategies. However, not all countries and regions are affected equally by drought. We fit a hierarchical Bayesian model with a dataset containing 60 years of country-level drought and agricultural productivity data to probabilistically identify the susceptibility of various countries and regions to drought. We find that regions such as Eastern Africa and Southern Asia are highly susceptible to drought, with each region exhibiting a >90% chance that drought has negatively affected agriculture, leading to estimated historical agricultural losses of >14%, while Eastern Asia is the most drought-resilient region, with only a 44% probability that drought has negatively affected agriculture in this region. The results of this study can help inform the allocation of future resources to enhance agricultural resilience in the most vulnerable regions. Additionally, they provide a foundation for case studies examining specific countries or regions that demonstrate notable resilience or susceptibility to drought.

## Introduction

As climate change intensifies, so too does the disruption to the global agricultural system. Mounting evidence highlights how anthropogenic climate change, along with other human-driven activities, has impacted water availability^1,2^ and increased the frequency or severity of extreme weather (e.g., droughts and heat waves)^3,4^, disrupting the agriculture sector in many parts of the world^5–7^. These findings have massive implications for both food security and economic stability for many countries^8,9^, highlighting the importance of understanding the relationship between climate change-driven events and global agriculture.

One of the most significant ways in which climate change affects agriculture is through the well-documented increase in frequency and severity of droughts in many regions^10,11^. Furthermore, climate change increases the likelihood of drought-related compound hazards including heat wave-drought co-occurrences^12–14^. There have been many case studies at the country^15–21^ and regional^22–26^ levels investigating the effects of drought on agricultural production, with many studies showing historical agricultural deficits attributable to drought, as well as predicting future agricultural losses.

Beyond case studies, additional macro-level analyses examining the role that drought has historically played in agricultural systems around the world have further elucidated the important relationship between drought and agriculture globally. In one example of such work, Lesk et al.^27^ use a superposed epoch analysis to estimate cereal production losses from the effects of drought and extreme heat, finding a 9-10% loss globally and relatively more losses in developed countries than developing countries. In a more recent work, Zaveri et al.^28^ use a fixed-effects regression analysis to generate a gridded world map of mean annual per capita GDP loss associated with drought. Although Zaveri et al. do not specifically study agricultural impacts, the authors find that much of the impact on GDP is caused by damage to agriculture^28^.

Here, we develop a hierarchical Bayesian regression model that provides, for the first time, country- and region-specific estimates of historical agricultural total factor productivity (TFP) deficits caused by drought, inclusive of model parameter uncertainty. This model facilitates comparing a given country’s or region’s likely range of responses to drought to the global average as well as to its geographic neighbors. Each nation’s agricultural industry has unique attributes that may affect its overall level of resilience to drought, such as the resources available to farmers in that country^29^, the economic prosperity of the country^30^, and the adaptation and long term planning abilities of the country’s institutions^31^, among many others. In addition to country-level differences, regional factors such as shared climate and weather patterns, similarity between types of crops grown in neighboring countries, and multi-country collaborative efforts to build resilience^32^ may also jointly impact the overall response to drought of groups of countries situated geographically near to each other, motivating the regional level analysis. As such, this work provides new insight into historical drought resilience at varying spatial scales, which may be useful in informing future policy decisions that are tailored for specific countries and regions.

The use of agricultural TFP growth as the dependent variable is based on prior work finding that it provides more consistent and robust results than using agricultural output^33^. Unlike many studies that identify extreme weather based on climate variables, we use the EM-DAT database^34^. The main difference between EM-DAT and other extreme weather indices is that EM-DAT identifies extreme weather based on direct human impact, while other approaches typically infer the presence of extreme weather based on a fixed definition, such as degree days above a threshold. The use of EM-DAT mitigates the bias and ambiguities potentially introduced by varying responses to high temperatures in different areas^27^. We include quadratic temperature in the model to isolate the effect of drought from other climate-related events, and include year-specific intercepts to capture global shocks in agriculture common to all countries. Our results are presented in terms of probabilities and likely ranges of values in order to avoid overconfidence in point estimates; these probabilities and confidence intervals stem from the coherent handling of model parameter uncertainty provided by the Bayesian framework.

## Results

### National results

**Fig. 1:**
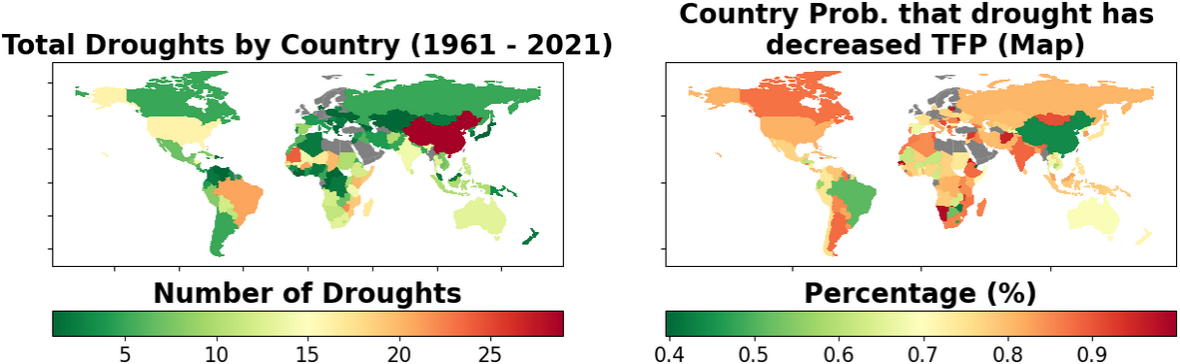
The leftward map shows the total number of droughts per country that have occurred in the 1961-2021 time range, according to the EM-DAT database^34^. The rightward map shows a geographical depiction of the probability that drought has decreased TFP in each country, based on the percentage of negative samples for each country-specific drought coefficient (see Methods Section). Countries with no recorded droughts in the database are shaded gray.

**Fig. 2:**
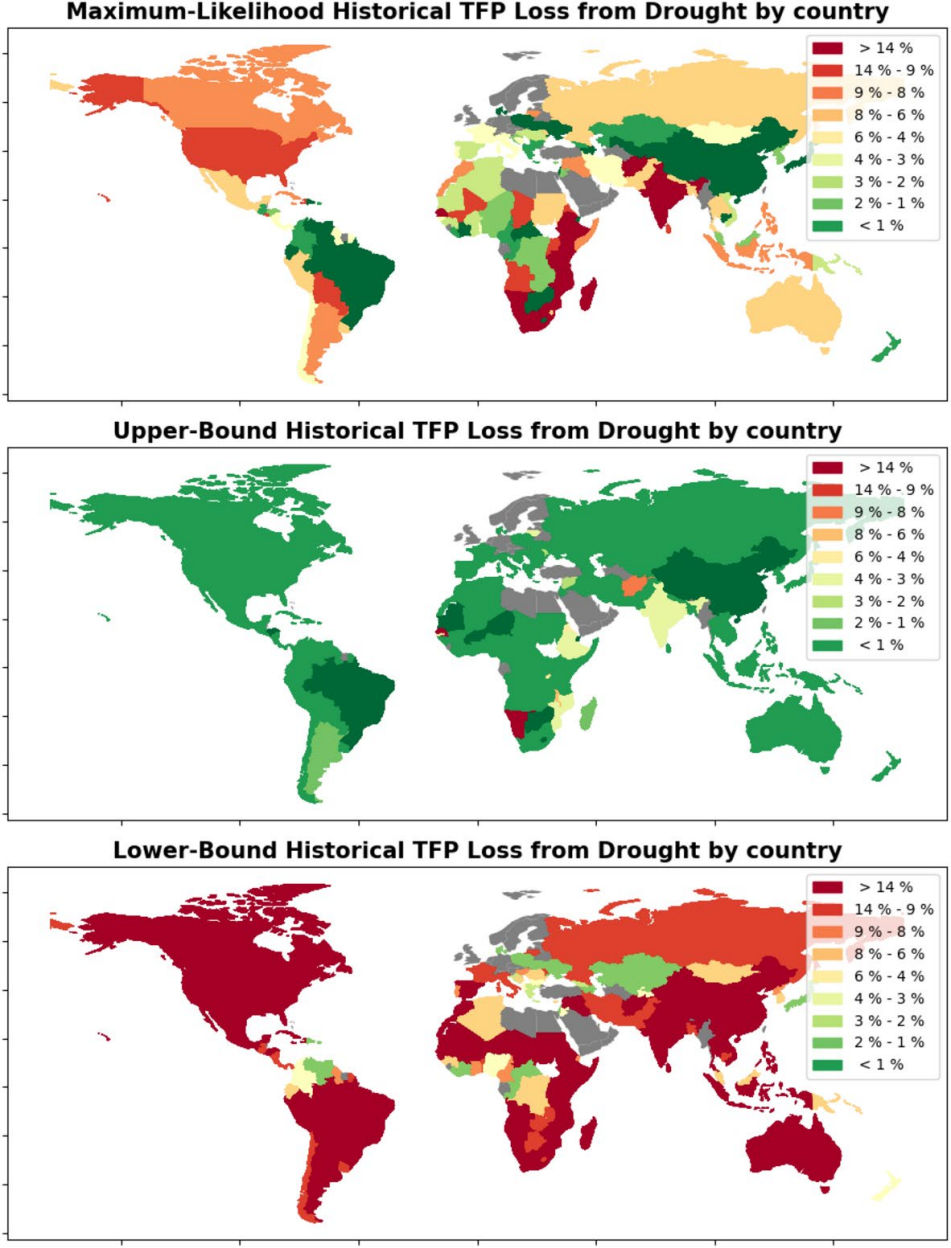
These three maps show the estimated percentage that drought has decreased TFP for each country. Estimates are provided as a lower and upper bound one standard deviation from the sampled mean, as well as a maximum likelihood estimate. The top map shows the maximum likelihood estimate for each country, which is computed by taking the mean of the range of estimates. The middle map shows the estimates at the 84th percentile of the range of estimates, meaning they form the upper bound of the range of estimates within one standard deviation of the mean. The bottom map shows the estimates at the 16th percentile, forming the lower bound of the same range of estimates within one standard deviation of the mean. Countries with no recorded droughts in the database are shaded gray.

The country-level results are presented in Figs. 1 and 2. In the lefthand side of Fig. 1, the total number of droughts per country are visualized, while on the righthand side, the probability that drought has negatively affected each country’s agriculture is visualized. The righthand side probabilities are based on the output of the Bayesian sampling; specifically, they reflect the percentage of drought coefficient samples that are negative for each country. For more details see the Methods section. Countries that have not experienced any drought in the EM-DAT database during the timeframe for which we have agricultural data are shaded in gray. Many of the world’s countries are shaded in orange or red indicating >80% probability that drought has negatively impacted agriculture. Overall, we see a high probability of negative drought impacts in the majority of the world’s countries, affirming the widely-held belief that increasing drought is indeed a major area of global concern for agriculture moving forward.

Interestingly, many of the countries in Fig. 1 exhibiting extreme results in one direction or the other are African nations. According to our model, two of the countries with the highest probability of drought negatively impacting agriculture are Senegal (99%) and Malawi (93%). The two countries with the lowest likelihood of negative agricultural impacts from drought are Djibouti (40%) and Zimbabwe (42%). Possible explanations include the potentially questionable reporting of agricultural data in these countries^35^, as well as the observation that smaller agricultural sectors are likely more sensitive to year-to-year changes, thus potentially leading to extreme results from our model. These results suggest that further work performing comparative studies between the agricultural sectors and agricultural data collection processes of various African nations might bear fruit in helping to explain the large differences in drought impacts found by our model in these countries.

In Fig. 2, the three maps show the maximum likelihood, upper bound, and lower bound estimates of the percentage that drought has negatively impacted agriculture for each country within the timeframe for which we have agricultural data (1961 - 2021). Upper- and lower-bounds reflect one standard deviation from the mean of the sampled drought coefficients for each country. Countries with no recorded drought during the timeframe of the analysis are again shaded in gray. One important takeaway from these maps is the large uncertainty in the model, indicated by the drastic shift from green to red, which many countries undergo between the upper- and lower-bound maps. This indicates that, at the country level, our model is providing wide ranges of likely percentage decreases from drought for many countries. Factors such as the quality of the agricultural data and the way that drought is modeled as a binary variable for each country/year observation may have caused uncertainty in our results to remain high; this topic will be revisited in the Discussion section.

Some countries present nearly uniform positive or negative responses to drought across the distribution of drought coefficient samples. For these countries, we can confidently conclude the effects of drought with high probability. For example, Trinidad and Tobogo, Liberia, Japan, Albania, and Nigeria each suffer only about a 5% or less decrease in TFP in our lower-bound estimate, indicating with high probability that drought has not severely impacted the agricultural sectors of these countries. On the other hand, based on our upper-bound estimates, there is high probability that drought seems to have delivered substantial losses to the agricultural sectors of countries such as Senegal (34% estimated upper-bound loss), Malawi (7%), and Afghanistan (8%). These observations reflect the utility of the hierarchical analysis for targeting specific countries particularly affected by drought; while there is no global solution to drought, resources can likely be used most efficiently by prioritizing the most vulnerable countries, which our model can help to identify. Supplementary Table S2 shows the estimated impacts of drought for all countries as a reference.

### Regional results

**Fig. 3:**
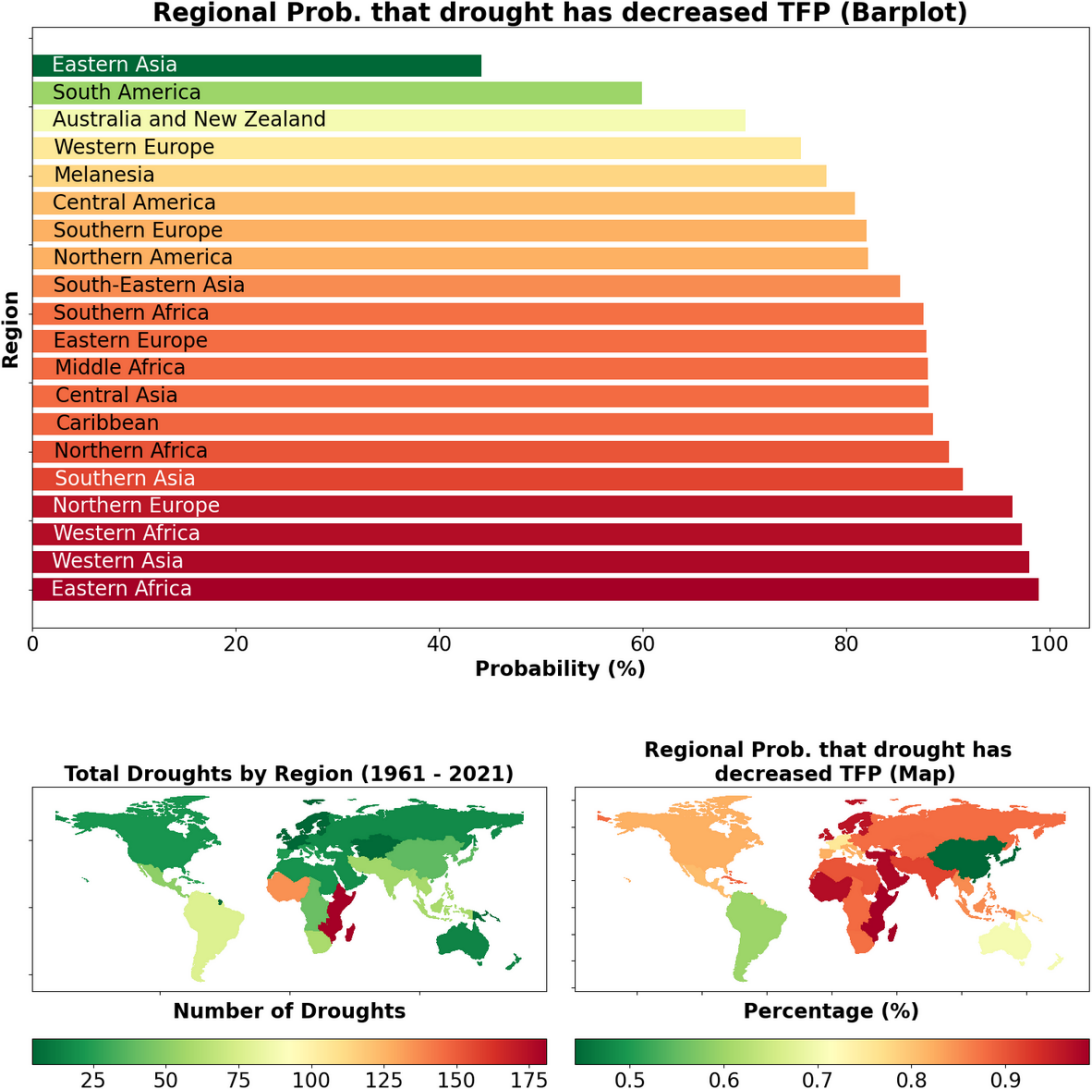
The barplot (top) shows the probability that drought has historically decreased agricultural TFP within the historical time period for which we have agriculture data (1961-2021), for each of the 20 world regions in our dataset. These probabilities are based on the percentage of negative samples for each regional slope. The map in the bottom left shows the total number of droughts per region that have occurred in the 1961-2021 time range, according to the EM-DAT database^34^. The map in the bottom right shows a geographical depiction of the same data as the barplot. Countries with no recorded droughts in the database are shaded gray.

**Fig. 4:**
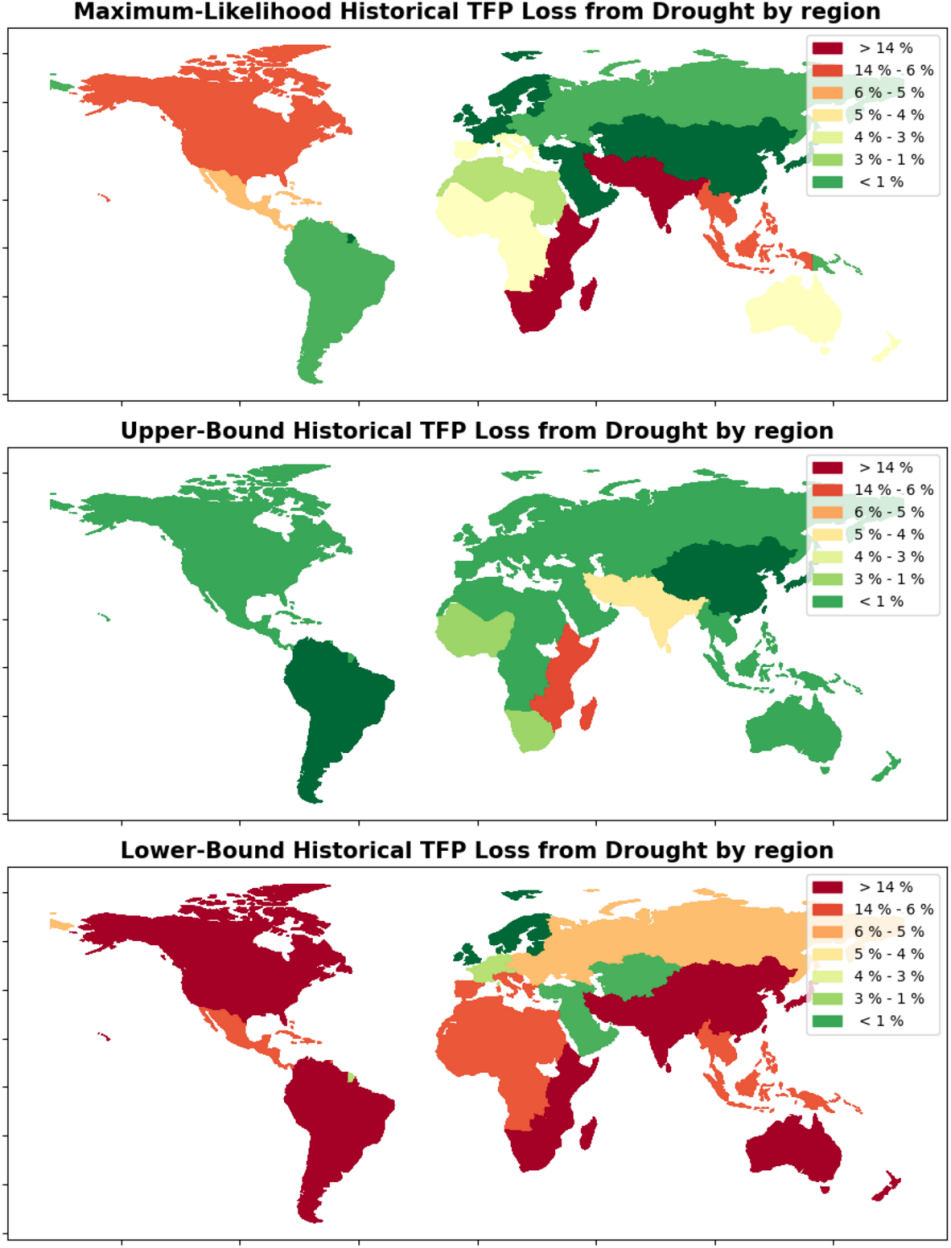
These three maps show the estimated percentage that drought has decreased TFP for each world region. As in Fig. 2, percentage loss estimates are provided as an upper bound and lower bound one standard deviation from the sampled mean, as well as a maximum likelihood estimate. Countries with no recorded droughts in the database are shaded gray.

The regional results of our analysis are presented in Figs. 3 and 4. The barplot at the top of Fig. 3 shows the probability that drought has negatively impacted the overall agricultural productivity for each of the 20 World Development Indicator regions in our dataset. The same data are shown as a map at the bottom right of the figure. The map at the bottom left of the figure shows the total number of droughts recorded by EM-DAT^34^ for each region in the time range for which agricultural TFP data is available.

Regional impacts are computed from the same model as the country impacts. To compute the regional impacts, the posterior distribution of the drought coefficient for each individual country is multiplied by that country’s regional share of agricultural production for each year that the country experienced drought, and these products are summed to create the posterior distribution of the total regional impact (see Section 4 for a more in-depth explanation). This methodology leads to more productive countries having more of an impact on the overall regional results than smaller ones, and “smooths out” some of the more heterogeneous country-level impacts seen in Fig. 2. For example, Botswana’s unexpected resilience to drought displayed by the model at the country level has been erased at the regional level, where we see that agriculture in the Southern Africa region appears highly drought-sensitive as a whole. While less granular than the country-level impacts, the regional impacts should be less susceptible to noisy or badly-collected data from individual countries, potentially providing a more robust perspective on overall drought impacts.

**Fig. 5:**
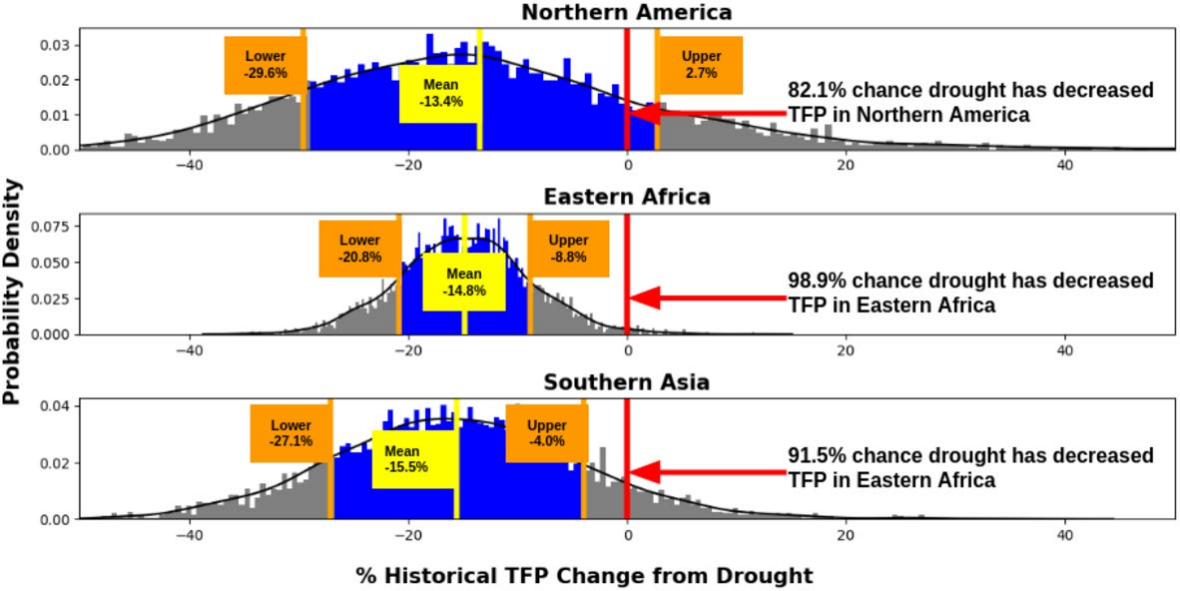
This figure shows the histograms of possible values for the percentage change in TFP due to drought in three world regions of concern. The horizontal yellow line in each histogram represents the mean, while the colored bins between the orange lines represent the range of values one standard deviation from the mean. The percentage likelihood that drought has decreased TFP is the percentage of samples below 0, or to the left of the red vertical line, for each region. Note that the yellow line representing the mean in each histogram corresponds to the maximum likelihood estimate in Fig. 4, while the rightward orange line in each histogram corresponds to the upper bound value and the leftward orange line corresponds to the lower bound.

Despite the reduction of country-level heterogeneity in response to drought, we still see that impacts of drought vary substantially between regions. Green regions South America and Eastern Asia appear relatively more resilient to drought, with the probability of drought having negatively affected agriculture around 60% and 44%, respectively for these regions. On the other hand, the reddest regions - Western Asia, Eastern Africa, Northern Europe, and Western Africa - all have probability greater than 97% that agriculture has been negatively affected by drought. One region of particular concern based on these results is Eastern Africa, which has a 99% of having been negatively affected by drought, likely in large part to having reported by far the most country-years in drought (181) of any region. Interestingly, Northern Europe, the region that has reported the fewest droughts (4), has one of the highest probabilities of having been negatively affected by drought. These results affirm previous findings that the African continent is among the most vulnerable to drought^36^, while Latin America is one of the more drought-resilient areas^27^.

Figure 4 shows three maps, which collectively show the maximum likelihood, upper bound, and lower bound estimates of the percentage decrease of TFP caused by drought for each region. The upper and lower bound estimates reflect one standard deviation from the mean of the distribution of samples for each country, while the maximum likelihood reflects the mean of the distribution. The Eastern Africa and Southern Asia regions appear to have suffered substantial declines in TFP from drought in all likelihood scenarios, with maximum likelihood estimate of 15% historical damage for both of these regions. In other world regions, the effect has been less drastic. For example, Northern Europe, a region that has experienced few droughts, is estimated to have lost only .7% of agricultural productivity due to drought. These maps provide a regional-level visualization of areas of concern, indicating that future work at the regional level should focus on the Eastern Africa, Southern Asia, and Northern America regions to help build drought resilience. Supplementary Table S2 shows the estimated impacts of drought for all regions as a reference.

In Fig. 5, we show the distribution of possible values of historical TFP change from drought for these three regions of concern, in the form of histograms. The yellow vertical lines show the means of the distributions, while the colored bins between the two orange lines show the range of values one standard deviation from the mean. Here, we see that the range of possible impacts in Northern America is quite large, indicating higher model uncertainty, while the variance of impacts in Eastern Africa is much smaller, indicating that the model is more certain about the impacts of drought in this region. The Southern Asia region exhibits higher uncertainty than Eastern Africa, but less than Northern America. By focusing on the variance in possible impacts rather than maximum likelihood estimates, we acknowledge that uncertainty is an inherent component of regression models, and emphasize the importance of being forthcoming regarding model parameter uncertainty, rather than ignoring it when convenient.

## Discussion

The results of this paper show a diverse array of historical impacts of drought on agricultural sectors at both the regional and national levels between the years 1961 - 2021. Although in this paper we focus specifically on droughts, our Bayesian methodology should be applicable to analyze the historical global impacts of any extreme weather event for which there is data available, as well as combinations of extreme weather events occurring simultaneously, including a coherent treatment of uncertainty.

We find that there are substantial differences between regions in the probability that drought has historically decreased agriculture, ranging from around 40% to nearly 100%. At the national level, we also see a wide range of responses between different countries. There are various possible factors that could cause this diversity of results between regions, such as the number and characteristics (intensity, duration) of droughts, the water requirements of particular crops grown in these areas, the climate of the regions, or adaptive measures taken by governments and perhaps individual farmers. The global response we compute estimates around a 5% global decrease in agriculture based on drought, with a 12% lower bound estimate (see Supplementary Figure S1).

Policy-driven drought management may play a large role in the heterogeneity of impacts observed at the country and regional levels. For example, Botswana has institutionalized its drought management at the level of the federal government and performs a rigorous drought assessment at the end of the rainy season every year^37^, which may help explain our study’s finding that it has been relatively drought-resilient compared to its neighbors. At the regional level, East African countries have failed to consistently implement drought mitigation policies, but could improve their agriculture’s resilience to drought via efforts to increase biodiversity and improve farm technology^38^. In future work, statistical analyses such as ours should be integrated with qualitative analyses of drought management policies, which will help to elucidate the relationship between drought management and the corresponding economic effects of drought in various countries and regions.

Further research may wish to use these results to perform within-region case studies comparing the agricultural conditions of regions and countries that appear particularly drought resilient, as well as neighboring countries that appear particularly susceptible. Of particular interest is the African continent, which is considered perhaps the most vulnerable continent to climate change generally^36^. In our models, Africa exhibits a diverse array of responses at both the regional and national levels, ranging from some of the most susceptible to the most resilient areas. Eastern Africa is the world region that has experienced the most drought and has suffered some of the most negative affects, with nearly all countries severely negatively impacted. However, Western Africa has also suffered a high number of droughts but includes countries, such as Nigeria and Liberia, that appear very resilient. In Southern Africa, a region that has suffered fewer droughts, Botswana and Zimbabwe appear to respond substantially better to droughts than neighbors South Africa and Mozambique. Further analysis of these neighboring countries exhibiting different responses to drought could perhaps help elucidate cross-border exchanges of ideas to promote unified regional responses using the best available techniques and knowledge.

The treatment of model parameter uncertainty facilitated by the Bayesian approach demonstrates the reality that drought’s impact on a given region or country is not always clear. While representing high model uncertainty in climate research may lead to communication challenges^39^, it is typically better than pretending uncertainty does not exist and presenting only the maximum likelihood estimates. Large uncertainty in climate econometric models is common, and presents the inconvenient reality that it is often difficult to be sure of the impact of the measured phenomenon on the target. While we can say with high probability that drought has decreased agricultural productivity globally based on our model results, there are many countries or regions for which we are much less certain of the historical effect on drought. For this reason, our results are better interpreted as general trends rather than hard estimates.

Along these lines, we recommend that our maximum-likelihood estimates be viewed as initial indicators of drought vulnerability and resilience, and contextualized with the explanation that they are means of a distribution with a certain variance, which can be presented alongside the mean estimates. These results are intended to be used as drivers of future research, rather than as a definitive and exact source of answers for where aid and resources should be targeted. Especially for national and regional drought responses exhibiting high uncertainty in our model, a within-country region-by-region analysis of agricultural response to drought (such as Chen et al.’s Bayesian approach to assessing regional drought response in China^26^) should be carried out before any definitive conclusions are drawn about a given country.

A limitation of the current work is that, unlike gridded drought data, the EM-DAT^34^ database’s treatment of drought as a binary variable for each country/year does not account for the scale of the droughts. In our model, a small portion of a country experiencing drought counts the same as if the entire land area of a country is in drought, even though these two scenarios will obviously impact agriculture differently. This problem may be especially apparent in larger countries like the United States and China. For example, a drought in California will have little effect on agriculture in New York, and may have led to high uncertainty about the effect of drought for these countries (and corresponding regions) in our model. Other factors unaccounted for in the EM-DAT^34^ data that may influence the effects of drought are whether the agricultural land is irrigated or rainfed^40,41^, and whether the drought occurred specifically over agricultural lands.

These factors likely play a role in our model’s high uncertainty for large countries such as China, even though past work using gridded drought and agricultural data has shown substantial negative impacts of drought on China’s agriculture^42,43^. However, most papers relying on gridded data have to grapple with how to define a drought based on climate variables, which may introduce bias by identifying droughts that did not have any real impact^27^. An advantage to the use of the EM-DAT database as our source of drought data is that droughts are identified purely based on human impact, which helps to mitigate this bias. It would be useful for future work to compare impacts of drought on agriculture using both gridded data and EM-DAT, inclusive of uncertainty, for each country and region.

## Methods

### Data sources

Our regression model investigates the global effect of drought on the first difference of the natural log of agricultural TFP. Using the hierarchical Bayesian approach, we simultaneously compute samples for a global slope for drought as well as a regional or country slope for drought. The regional definitions come from the 20 world regions present in our dataset, as defined by the World Bank Development Indicators. We obtain agricultural TFP data from the United States Department of Agriculture (USDA) Economic Research Service (ERS)^44^ and data on droughts from the Emergency Events Database (EM-DAT)^34^. This database marks emergency disasters that have led to one of at least 100 people affected, 10 fatalities, a declaration of emergency, or a call for international assistance. The model also includes quadratic annual average temperature weighted by agricultural land as an additional explanatory variable. Temperature and precipitation data for each country year is extracted from the gridded temperature data made available by the NCEP/NCAR 40-year reanalysis project^45^, and we use the agricultural dataset available at www.earthstat.org^46^ to compute weights for climate variables.

### Model training

We trained a hierarchical Bayesian model to learn the relationship between drought and TFP growth at the country level. Note that in other literature, hierarchical models are sometimes also referred to as both multi-level models and random slopes models. Drought is represented as a binary variable for each country year, in which a 1 represents that EM-DAT recorded at least one drought somewhere in the country during the specified year. The model also includes quadratic annual average temperature weighted by agricultural land to account for non-drought-related effects of climate on agriculture, and year-specific fixed effects to account for global events that affected all countries.

Based on its hierarchical nature, the model samples country-specific coefficient values for drought based on a single, global distribution of coefficient values which is learned simultaneously as part of a joint multivariate distribution, allowing for variance in the country-specific outcomes based on locality-specific policies and adaptations. The set of equations for the hierarchical model is shown below in Eqs. (1) to (2).

1 $$\begin{aligned} y_{it}\sim & \mathcal {N}(u_{it}, HN(\theta _0)) \end{aligned}$$

2 $$\begin{aligned} u_{it}= & \beta _{0t} + (\beta _{1i} * D_{it}) + \sum _{j=2}^{k} (\beta _{j} * C_{it}) \end{aligned}$$

3 $$\begin{aligned} \begin{pmatrix}\beta _{0t}\\ \beta _{1i}\\ \beta _j-\beta _k\\ \end{pmatrix}\sim & \mathcal {N} \begin{bmatrix} \begin{pmatrix} \mu _{0}\\ \mathcal {N}(_{\mathcal {N}(\mu 1,\sigma _1)i}, HN(\theta _1))\\ \mu _2\\ \end{pmatrix}\!\!, \begin{pmatrix} \sigma _0\\ HN(\theta _2)\\ \sigma _2\\ \end{pmatrix} \end{bmatrix} \end{aligned}$$

Equation (1) shows the outcome *y* (first difference of the natural log of agricultural TFP) for each i (country) and t (year) represented as a Normal distribution ($\mathcal {N}$) parameterized by a mean $\mu$ which is the sum of the regression terms in Eq. (2) as well as a variance term. Equation (2) shows the sum of the regression terms, where $\beta _0$ represents the year-specific intercepts, $\beta _1$ represents the country–specific coefficient for drought D, and $\beta _k$ - $\beta _j$ represent the coefficients for C, the matrix of climate covariates, which are *Temperature* and $Temperature^2$. Equation (2) shows that the year-specific intercepts $\beta _0$ and the model coefficients $\beta _1$ - $\beta _4$ are drawn from normal distributions. Coefficients $\beta _1$ for drought in a given country i represent the hierarchical component. The mean of $\beta _{1i}$ is drawn from a normal distribution for each country whose mean is in turn drawn from a global distribution of coefficients. To allow for the model to flexibly learn the variance of the global and country-level distributions for drought, the variance term for these distributions are Half Normal (*HN*) distributions parameterized by scale terms $\theta _1$ and $\theta _2$.

We used uninformative priors to allow the data to guide the sampler, which are displayed in Supplementary Table S1. The code for the model is written in PyMC^47^ and sampling is performed using the NUTS algorithm^48^. We sampled across 4 Markov Chain Monte Carlo chains and burn-in 5000 samples and save 5000 samples per chain, for a total of 20,000 posterior samples.

### Robustness checks

In order to assert the robustness to permutation of the model shown in Eqs. (1) - (2), we fit two additional hierarchical models with different climate covariates. The first additional model includes no climate variables (with drought as the only covariate), and the second additional model includes quadratic temperature and quadratic precipitation as covariates (*Temperature*, $Temperature^2$, *Precipitation*, $Precipitation^2$). Like our primary model, both model variations include year-specific fixed effects. The country-specific drought coefficient means learned in each of these models were quite similar to those learned in the primary model, indicating that our model is robust against different permutations of climate covariates. The drought coefficients for each model are shown in Supplementary Table S4.

As an additional robustness check, we fit a model using vegetation coverage, rather than agricultural productivity, as the dependent variable. The purpose of doing so was to help address data quality concerns regarding the USDA agricultural productivity data we used in our primary analysis. We used the PKU Normalized Difference Vegetation Index (NDVI) dataset^49^ for this purpose. The drought coefficients for this model are also shown in the last column of Supplementary Table S4. We observed that the coefficient means are mostly similar between this model and the other three models using the USDA data, indicating that our results are not simply a product of the selected agricultural dataset. However, there are some notable exceptions which might indicate concerns about the validity of the findings for certain countries. For example, countries such as Afghanistan, Botswana, and Djibouti exhibited a much more negative response to drought using the NDVI data than the USDA data, which may indicate questionable agricultural data for these countries. It might also indicate that NDVI is not a good proxy for agricultural impacts in these countries, since natural vegetation and cropland may respond differently to drought.

### Computing impacts

After training the models, we obtain the probability that drought has decreased TFP within the timeframe spanned by our data source in each country by observing the percentage of samples for the drought coefficient for that country that are below 0, which provides the data for the right panel of Fig. 1.

Percentage losses for each country is computed by carrying out the following steps:

1. Our model provides a country-specific posterior distribution representing the impact of a single drought on agricultural productivity.
2. This distribution is multiplied by the total number of droughts that the country has experienced in the observed time period to obtain distributions for the change in the natural log of TFP based on drought for each country.
3. The resulting distribution is converted into a percentage change by unlogging the value (raising *e* to the value of the total), subtracting 1, and multiplying by 100.

Since the posterior distribution of each country-specific coefficient is comprised of many samples, this procedure leads to a range of possible percentage loss estimates for each country, which provides the data for Fig. 2.

We also compute impacts at higher levels using three country groupings: by region, the entire globe (for Supplementary Figure S1), and by UN development group (for Supplementary Figure S2). We obtain country weights from the share of each country’s agricultural revenue in the larger group, using data from the United States Department of Agriculture^36^. Then, for the countries in each group (which, for the global group, includes all countries), we execute the following steps to compute group-level impacts:

1. For each country, the posterior distribution of the drought coefficient is multiplied by the country weight, which represents the share of agricultural revenue that the country holds with respect to the larger group.
2. The resulting distribution is multiplied by the number of droughts that the country has experienced in the observed time period.
3. These distributions are then summed across all countries in the region to provide distributions for the total change in natural log of TFP from drought for the region.
4. This total is converted into a percentage in the manner described in Step 3 in the previous list.

This procedure provides the region-specific data for Figs. 3 and 4. The data from the barplot and bottom right panel of Fig. 3 represents the number of samples from the regional distributions that are below 0. In Fig. 5, we show three selected regional impact estimates as histograms rather than as a map, using the same regional estimates as in the map figure.

## Supplementary Information

Supplementary Information.

## Data Availability

The data and code for the models and data processing is available at https://github.com/greenguy33/hierarchical_bayesian_drought_study_code.
