# Supplementary material for: Disparities in the impact of drought on agriculture across countries
Source: Sci Rep. 2025 Apr 18;15:13465. doi: 10.1038/s41598-025-94166-z (PMC12008377; doi:10.1038/s41598-025-94166-z)
Supplement: Supplementary file 1 — Supplementary Information. [file 41598_2025_94166_MOESM1_ESM.pdf]

Supplementary Information.

S1 Bayesian Model Priors

.
Table S1 This table shows the uninformative Bayesian priors used as inputs to the MCMC sampling algorithm. We purposefully use large priors to allow the data to speak for itself and let the model converge to the appropriate value. This approach is likely to give the same (or very similar) maximum-likelihood estimates as a frequentist regression model, but with the addition of posterior samples that can be used to compute confidence bounds. Symbols from this table are referenced from equations 1 - 3. Hyperparameters are defined as parameters of prior distributions, and hyperpriors are the distributions parameterized by hyperparameters.

Hyperparameter Symbol	Hyperparameter Description	Prior Value
μ_0	Mean of Normal Distributions for year-specific intercepts	0
μ_1	Mean of Normal Distribution for global drought coefficient	0
μ_2	Mean of Normal Distributions for climate covariates	0
θ_0	Scale of HalfNormal Distribution for dependent variable (TFP growth) hyperprior distribution	10
θ_1	Scale of HalfNormal Distribution for global drought coefficient standard deviation	10
θ_2	Scale of HalfNormal Distribution for country-level drought coefficient standard deviations	10
σ_0	Standard deviation of Normal Distribution for year-specific intercepts	5
σ_1	Standard deviation of Normal Distribution for global drought coefficient hyperprior	10
σ_2	Standard deviation of Normal Distribution for climate covariate coefficients	5

S2 Global Results

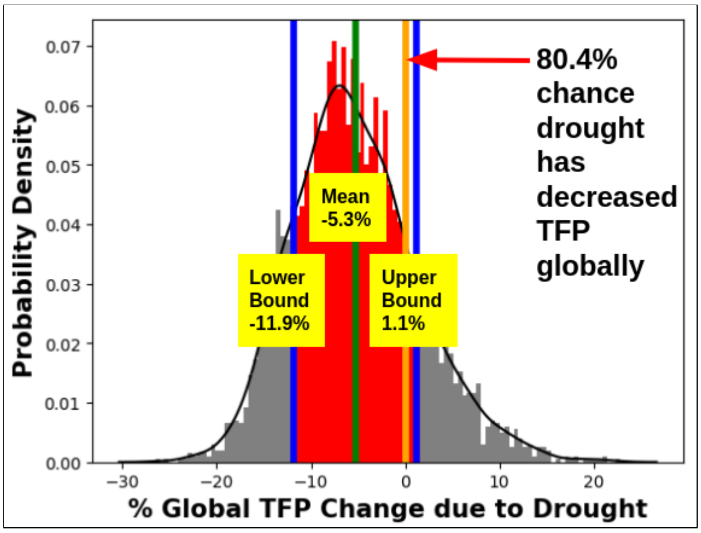

Figure S1 Histogram showing the estimated global percentage decrease of TFP to drought between the years 1961 - 2021, generated from samples of the global drought coefficient from the regional hierarchical model. This model estimates the global agricultural deficit from drought to have a maximum likelihood of a 5.3%, with lower bound one standard deviation from the mean of 11.9%. 80.4% of samples fall below 0, indicating our model's estimated probability that drought has decreased agriculture globally.

Supplementary Figure S1 shows a histogram plot of the likely range of historical global TFP loss due to drought. At the global level, our model returns a maximum likelihood estimate of a 5% historical reduction in agriculture due to drought between 1961 and 2021, and estimates a 80% likelihood that drought has indeed decreased global agriculture. These estimates are computed by aggregating the sampled drought responses of individual countries weighted by their respective market share of global agriculture. The percentage likelihood of a global decrease in agriculture due to drought is the percentage of possible estimates that are below 0. These results show a somewhat mixed picture of the global effect of drought on agriculture; while they indicate that it is most likely that drought has decreased agriculture globally,there is enough model parameter uncertainty that the possibility that drought has not decreased global agricultural productivity at all is within the likely range.

Our global lower bound estimate broadly aligns with the estimate of Lesk et al. [27] of between 9% and 10% global cereal production loss between the years of 1964 - 2007, although their analysis also factors in the agricultural damage caused by extreme heat, and also uses a different statistical approach to ours.

S3 Developed vs. Developing Countries

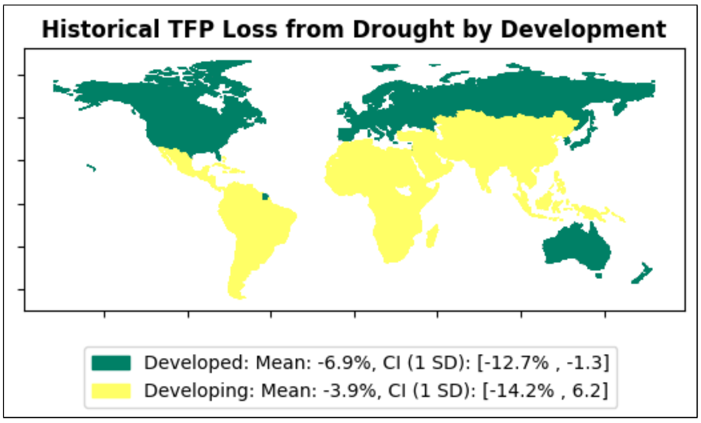

Figure S2 Map showing the developed vs. developing world according to United Nations Trade and Development [50] alongside mean estimates and confidence intervals for each group. Although the mean estimate of percentage agricultural damages is higher for the developed world, wider confidence intervals for the developing world imply less certainty about the result.

To further relate our work to the analyses performed by Lesk et al. [27] and Zaveri et al. [28], we compute the historical TFP losses for two groups of countries comprising developed and developing nations. To define the groups, we use the definition provided by United Nations Trade and Development [50]. Our results, shown in Supplementary Figure S2, indicate that, while the maximum likelihood estimate (mean) of the developed group implies greater losses (7% total losses across all countries in the group) than the developing group (4% total losses), the developing group's CI encompasses the entire CI of the developed group, indicating greater uncertainty in the developing group. For this reason, we cannot concretely conclude whether agriculture in the developing or developed group has been damaged more by drought, although based on the maximum likelihood estimates, the developed group has been harmed more, which aligns with the findings of Lesk et al. [27].

S4 Agricultural Revenues by Country

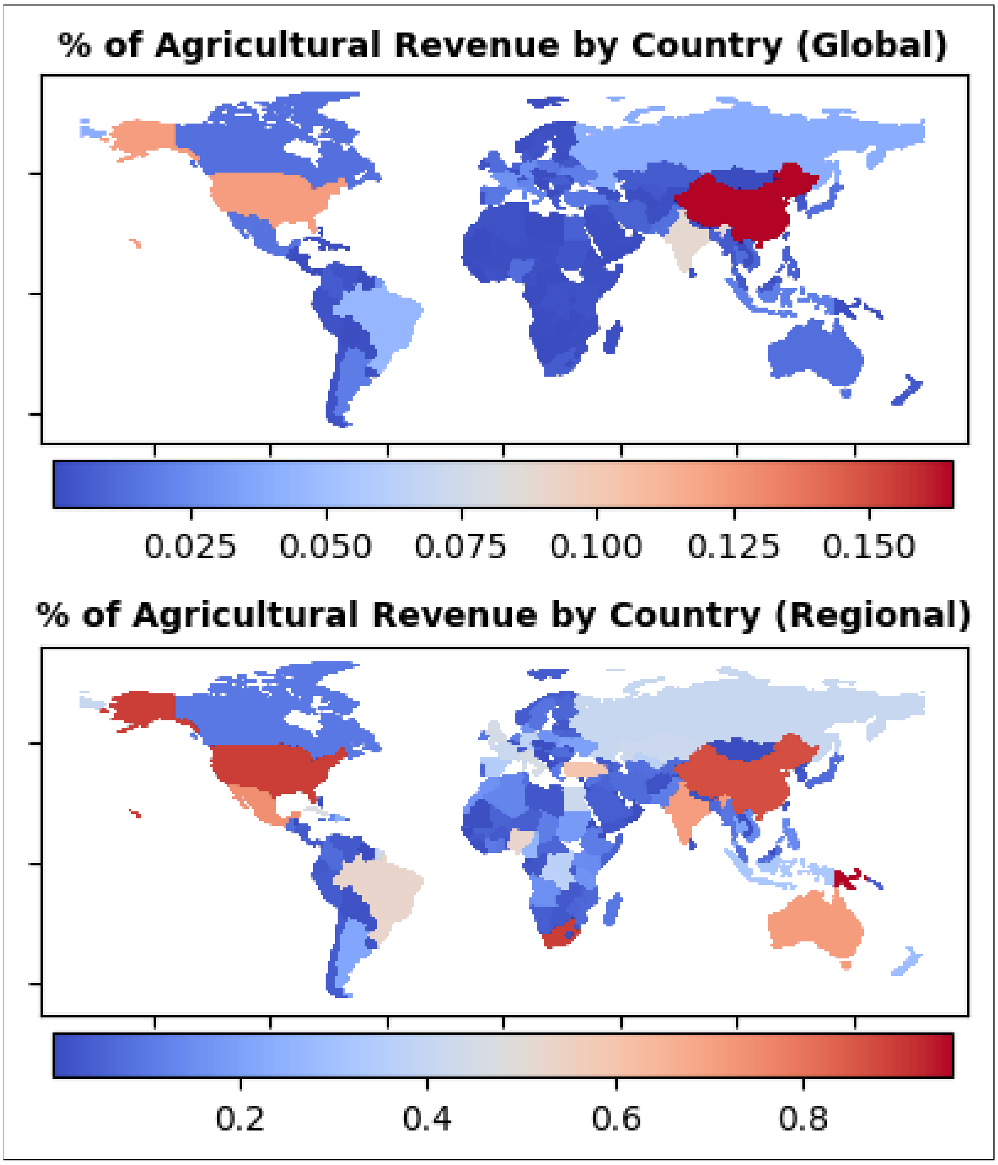

Figure S3 This figure shows global- (top) and regional-level (bottom) percentages of agricultural revenue by country. These percentages are used as weights for the computation of regional- (Figures 3, 4) and global-level (Supplementary Figure S1) historical change in TFP due to drought. Countries with a higher percentage of regional percentage of agricultural revenue are weighted higher in the regional-level analysis, leading to some smoothing of the results compared to the country level, where countries with smaller (and potentially more sensitive) agricultural sectors likely cause the model to find a wide variety of drought responses within the same region, particularly in Africa (see Figure 2). The agricultural revenue percentages are computed based on data provided by the United States Department of Agriculture [36], and the region definitions are provided by the World Bank Development Indicators.

S5 Regional Drought Impacts Summary

Table S2: Summary statistics for regional drought impacts

Region Name	% Region Impact Samples < 0	Historical % TFP Change from Drought Mean	Historical % TFP Change from Drought SD
Australia and New Zealand	70.100	-5.246	12.221
Caribbean	88.525	-6.526	5.723
Central America	80.825	-5.981	7.458
Central Asia	88.100	-1.120	1.006
Eastern Africa	98.900	-14.832	5.993
Eastern Asia	44.125	10.997	35.780
Eastern Europe	87.900	-3.442	3.181
Melanesia	78.025	-3.126	4.507
Middle Africa	88.050	-5.145	4.314
Northern Africa	90.100	-3.746	3.109
Northern America	82.125	-13.430	16.153
Northern Europe	96.375	-0.712	0.449
South America	59.900	-1.767	14.585
South-Eastern Asia	85.275	-6.631	6.404
Southern Africa	87.600	-14.841	13.548
Southern Asia	91.500	-15.524	11.568
Southern Europe	82.000	-4.461	5.302
Western Africa	97.275	-5.211	2.661
Western Asia	98.025	-1.556	0.851
Western Europe	75.575	-1.736	2.670

S6 Country Drought Coefficient and Impacts Summary

Table S3: Summary statistics for national drought coefficients and impacts

ISO3 Country Code	Drought Coef. Mean	Drought Coef. SD	% Drought Coef Samples < 0	Historical % TFP Change from Drought Mean	Historical % TFP Change from Drought SD
AFG	-0.023	0.015	95.275	-19.629	11.980
AGO	-0.010	0.014	78.025	-10.072	14.861
ALB	-0.009	0.017	73.050	-1.711	3.336
ARG	-0.017	0.016	88.000	-7.974	7.321
ARM	-0.013	0.017	80.075	-1.247	1.646
AUS	-0.006	0.013	68.875	-6.235	16.901
AUT	-0.012	0.018	76.750	0.000	0.000
AZE	-0.012	0.017	77.300	-1.133	1.693
BDI	-0.023	0.017	94.225	-14.471	9.655
BEL	-0.011	0.018	76.975	0.000	0.000
BEN	-0.006	0.015	68.075	-2.740	7.597
BFA	-0.003	0.012	62.600	-3.834	25.714
BGD	-0.013	0.016	82.225	-6.069	7.410
BGR	-0.009	0.017	73.000	-1.679	3.304
BHR	-0.012	0.017	76.675	0.000	0.000
BHS	-0.012	0.018	77.425	0.000	0.000
BIH	-0.021	0.018	90.825	-4.069	3.416
BLR	-0.012	0.018	77.000	0.000	0.000
BLZ	-0.012	0.018	77.175	0.000	0.000
BOL	-0.013	0.014	85.300	-13.765	14.066
BRA	0.000	0.013	51.050	4.317	30.002
BRN	-0.012	0.017	77.000	0.000	0.000
BTN	-0.012	0.017	77.850	0.000	0.000
BWA	0.000	0.014	52.475	1.452	16.719
CAF	-0.011	0.017	77.350	-1.122	1.692
CAN	-0.017	0.016	87.250	-7.646	7.179

Continued on next page

ISO3 Country Code	Drought Coef. Mean	Drought Coef. SD	% Drought Coef Samples ¡ 0	Historical % TFP Change from Drought Mean	Historical % TFP Change from Drought SD
CHE	-0.012	0.017	77.600	0.000	0.000
CHL	-0.010	0.015	75.300	-4.564	7.365
CHN	0.002	0.012	43.925	13.836	42.665
CIV	-0.012	0.018	77.475	-1.152	1.760
CMR	-0.004	0.016	62.500	-1.611	7.915
COD	-0.014	0.017	81.100	-2.689	3.277
COG	-0.013	0.017	80.375	-1.323	1.672
COL	-0.010	0.017	74.150	-1.831	3.248
CRI	-0.012	0.015	80.175	-5.444	7.265
CUB	-0.011	0.015	79.050	-7.931	10.926
CYP	-0.014	0.016	82.375	-2.680	3.124
CZE	-0.012	0.018	76.350	0.000	0.000
DEU	-0.012	0.018	77.525	0.000	0.000
DJI	0.005	0.016	39.525	5.786	15.451
DNK	-0.012	0.017	77.625	-1.166	1.722
DOM	-0.012	0.018	77.200	-1.143	1.747
DZA	-0.017	0.017	85.075	-3.205	3.373
ECU	-0.001	0.016	57.075	-0.261	6.635
EGY	-0.012	0.017	77.075	0.000	0.000
ERI	-0.027	0.018	96.600	-12.370	7.554
ESP	-0.005	0.014	66.975	-3.966	12.749
EST	-0.012	0.018	76.900	0.000	0.000
ETH	-0.016	0.014	88.050	-16.414	14.195
FIN	-0.012	0.018	77.525	0.000	0.000
FRA	-0.011	0.016	75.575	-3.998	6.263
GAB	-0.012	0.017	78.950	0.000	0.000
GBR	-0.012	0.017	78.275	0.000	0.000
GEO	-0.018	0.018	86.575	-1.783	1.753
GHA	-0.010	0.016	73.575	-3.555	6.220
GIN	-0.011	0.016	77.300	-3.051	4.742
GMB	-0.015	0.014	87.950	-17.960	15.559
GNB	-0.007	0.015	70.850	-5.064	11.422
GNQ	-0.012	0.017	76.600	0.000	0.000
GRC	-0.017	0.018	85.150	-1.705	1.739
GTM	-0.003	0.015	60.675	-1.597	12.385
GUF	-0.012	0.018	78.100	0.000	0.000
GUY	-0.016	0.016	86.900	-4.710	4.538
HND	-0.003	0.014	61.650	-2.296	19.323

Continued on next page

ISO3 Country Code	Drought Coef. Mean	Drought Coef. SD	% Drought Coef Samples ¡ 0	Historical % TFP Change from Drought Mean	Historical % TFP Change from Drought SD
HRV	-0.021	0.018	90.275	-2.051	1.755
HTI	-0.012	0.014	81.450	-11.167	13.689
HUN	-0.013	0.017	79.500	-3.632	4.839
IDN	-0.010	0.014	78.400	-8.866	12.933
IND	-0.016	0.014	88.525	-18.375	15.609
IRL	-0.011	0.018	76.325	0.000	0.000
IRN	-0.013	0.017	80.075	-4.718	6.276
IRQ	-0.016	0.016	85.650	-8.694	8.603
ISR	-0.012	0.017	78.575	-1.183	1.652
ITA	-0.014	0.016	81.600	-5.102	6.171
JAM	-0.017	0.016	87.275	-7.985	7.287
JOR	-0.011	0.017	76.700	-2.081	3.253
JPN	-0.010	0.017	74.400	-0.976	1.715
KAZ	-0.013	0.017	79.800	-1.270	1.681
KEN	-0.010	0.012	81.150	-15.914	20.318
KGZ	-0.013	0.017	79.750	-1.233	1.647
KHM	-0.002	0.016	58.325	-0.703	11.159
KOR	-0.015	0.017	83.200	-2.961	3.225
KWT	-0.012	0.018	78.325	0.000	0.000
LAO	-0.010	0.015	76.550	-6.357	10.042
LBN	-0.012	0.017	78.425	0.000	0.000
LBR	-0.014	0.017	81.600	-1.336	1.672
LBY	-0.012	0.017	77.875	0.000	0.000
LKA	-0.010	0.014	78.975	-10.581	14.810
LSO	0.002	0.015	47.425	3.844	17.201
LTU	-0.029	0.019	96.375	-8.141	5.162
LUX	-0.012	0.017	77.525	0.000	0.000
LVA	-0.013	0.018	79.150	0.000	0.000
MAR	-0.016	0.015	86.300	-8.588	8.263
MDA	-0.026	0.018	94.775	-7.302	4.989
MDG	-0.013	0.013	85.850	-18.025	18.075
MEX	-0.010	0.015	77.200	-6.306	9.683
MKD	-0.021	0.018	90.350	-2.037	1.724
MLI	-0.010	0.014	77.575	-11.016	17.318
MLT	-0.012	0.017	78.000	0.000	0.000
MMR	-0.012	0.018	76.550	0.000	0.000
MNG	-0.021	0.018	90.625	-4.090	3.404
MOZ	-0.014	0.012	86.900	-20.675	18.997

Continued on next page

ISO3 Country Code	Drought Coef. Mean	Drought Coef. SD	% Drought Coef Samples ¡ 0	Historical % TFP Change from Drought Mean	Historical % TFP Change from Drought SD
MRT	-0.003	0.012	61.550	-3.213	31.040
MWI	-0.020	0.015	92.750	-18.568	13.033
MYS	-0.015	0.017	82.550	-2.879	3.291
NAM	-0.032	0.017	98.675	-28.646	12.873
NER	-0.003	0.013	61.675	-2.396	22.979
NGA	-0.012	0.017	77.000	-2.237	3.243
NIC	-0.008	0.015	72.150	-4.533	8.860
NLD	-0.012	0.018	76.625	0.000	0.000
NOR	-0.012	0.017	78.150	0.000	0.000
NPL	-0.014	0.015	83.725	-7.553	8.210
NZL	-0.008	0.017	71.450	-1.601	3.291
OMN	-0.012	0.017	78.475	0.000	0.000
PAK	-0.012	0.015	81.025	-5.761	7.060
PAN	-0.012	0.016	78.575	-4.335	5.971
PER	-0.009	0.015	74.575	-5.957	11.245
PHL	-0.009	0.014	75.250	-8.233	14.653
PNG	-0.011	0.016	77.525	-3.113	4.677
POL	-0.012	0.016	77.150	-1.132	1.623
PRI	-0.014	0.017	82.150	-1.378	1.630
PRK	-0.006	0.016	66.000	-2.051	6.382
PRT	-0.016	0.016	85.825	-4.595	4.551
PRY	-0.012	0.014	82.100	-11.691	13.836
PSE	-0.012	0.017	78.250	0.000	0.000
QAT	-0.012	0.018	77.725	0.000	0.000
ROU	-0.016	0.017	85.600	-3.160	3.252
RUS	-0.012	0.016	80.650	-5.739	7.375
RWA	-0.013	0.014	83.450	-10.332	11.516
SAU	-0.012	0.017	77.325	0.000	0.000
SDN	-0.009	0.015	74.200	-7.393	13.757
SEN	-0.039	0.016	99.950	-49.779	14.722
SLB	-0.011	0.016	78.300	-3.236	4.545
SLE	-0.012	0.018	76.925	0.000	0.000
SLV	-0.015	0.015	86.450	-9.472	9.377
SOM	-0.006	0.013	69.225	-7.616	23.555
SUR	-0.012	0.017	78.625	0.000	0.000
SVK	-0.012	0.018	76.925	0.000	0.000
SVN	-0.012	0.018	77.225	0.000	0.000
SWE	-0.012	0.017	78.825	0.000	0.000

Continued on next page

ISO3 Country Code	Drought Coef. Mean	Drought Coef. SD	% Drought Coef Samples ¡ 0	Historical % TFP Change from Drought Mean	Historical % TFP Change from Drought SD
SWZ	-0.006	0.013	68.400	-5.710	16.913
SYR	-0.023	0.017	93.025	-8.548	6.179
TCD	-0.009	0.012	77.475	-13.400	21.479
TGO	-0.014	0.016	82.475	-3.998	4.665
THA	-0.006	0.014	67.475	-5.647	17.450
TJK	-0.006	0.016	67.450	-1.189	3.236
TKM	-0.012	0.017	77.350	0.000	0.000
TTO	-0.010	0.017	73.800	-0.950	1.690
TUN	-0.019	0.017	89.175	-3.759	3.286
TUR	-0.012	0.018	76.925	0.000	0.000
TWN	-0.012	0.018	78.175	0.000	0.000
TZA	-0.013	0.013	84.625	-14.954	15.400
UGA	-0.014	0.014	84.850	-12.185	12.538
UKR	-0.011	0.017	76.200	-1.112	1.728
URY	-0.014	0.016	84.025	-6.700	7.228
USA	-0.011	0.013	81.225	-13.850	17.896
UZB	-0.011	0.017	77.675	-1.125	1.669
VEN	-0.012	0.017	79.025	-1.214	1.668
VNM	-0.005	0.015	66.400	-3.519	12.021
YEM	-0.011	0.018	77.725	0.000	0.000
ZAF	-0.013	0.013	85.775	-14.776	15.055
ZMB	-0.004	0.016	63.050	-2.038	11.028
ZWE	0.004	0.013	41.700	12.491	33.566

S7 Country Drought Coefficient by Model Variation

Table S4: Mean of the posterior distribution of the drought coefficient for each of the four models used for robustness checks. Model0 contained drought as the only covariate, without any additional climate covariates. Model1 contained drought and quadratic temperature as covariates, and was used to generate this paper's results. Model2 contained drought, quadratic temperature, and quadratic precipitation as covariates. Model3 used NDVI data as the dependent variable and contained drought and quadratic temperature as covariates.

ISO3 Country Code	Model0 Drought Coef. Means	Model1 Drought Coef. Means	Model2 Drought Coef. Means	Model3 Drought Coef. Means
AFG	-0.021	-0.023	-0.022	-0.046
AGO	-0.010	-0.010	-0.010	-0.010
ALB	-0.010	-0.009	-0.008	-0.011
ARG	-0.016	-0.017	-0.016	-0.017
ARM	-0.011	-0.013	-0.013	-0.016
AUS	-0.006	-0.006	-0.006	-0.019
AUT	-0.013	-0.012	-0.012	-0.014
AZE	-0.012	-0.012	-0.011	-0.014
BDI	-0.021	-0.023	-0.022	-0.007
BEL	-0.012	-0.011	-0.012	NaN
BEN	-0.008	-0.006	-0.007	-0.009
BFA	-0.006	-0.003	-0.003	-0.013
BGD	-0.014	-0.013	-0.013	-0.007
BGR	-0.010	-0.009	-0.009	-0.012
BHR	-0.010	-0.012	-0.011	NaN
BHS	-0.013	-0.012	-0.011	-0.014
BIH	-0.020	-0.021	-0.020	NaN
BLR	-0.011	-0.012	-0.012	-0.014
BLZ	-0.011	-0.012	-0.011	-0.014
BOL	-0.014	-0.013	-0.013	-0.015
BRA	-0.000	0.000	-0.001	-0.008
BRN	-0.012	-0.012	-0.011	-0.014
BTN	-0.011	-0.012	-0.011	-0.014
BWA	-0.000	0.000	0.000	-0.038
CAF	-0.012	-0.011	-0.011	-0.010
CAN	-0.017	-0.017	-0.016	-0.010
CHE	-0.012	-0.012	-0.011	-0.014
CHL	-0.009	-0.010	-0.009	-0.012
CHN	0.001	0.002	0.002	-0.005

Continued on next page

ISO3 Country Code	Model0 Drought Coef. Means	Model1 Drought Coef. Means	Model2 Drought Coef. Means	Model3 Drought Coef. Means
CIV	-0.011	-0.012	-0.011	-0.011
CMR	-0.005	-0.004	-0.004	-0.011
COD	-0.013	-0.014	-0.013	-0.011
COG	-0.012	-0.013	-0.013	-0.014
COL	-0.011	-0.010	-0.009	-0.012
CRI	-0.014	-0.012	-0.012	-0.015
CUB	-0.011	-0.011	-0.011	-0.014
CYP	-0.014	-0.014	-0.013	-0.021
CZE	-0.012	-0.012	-0.011	NaN
DEU	-0.012	-0.012	-0.011	-0.014
DJI	0.002	0.005	0.005	-0.022
DNK	-0.012	-0.012	-0.011	-0.013
DOM	-0.012	-0.012	-0.011	-0.014
DZA	-0.016	-0.017	-0.015	-0.014
ECU	-0.003	-0.001	-0.003	-0.014
EGY	-0.012	-0.012	-0.011	-0.014
ERI	-0.025	-0.027	-0.025	NaN
ESP	-0.007	-0.005	-0.006	-0.010
EST	-0.011	-0.012	-0.012	-0.014
ETH	-0.015	-0.016	-0.016	NaN
FIN	-0.011	-0.012	-0.012	-0.014
FRA	-0.012	-0.011	-0.010	-0.012
GAB	-0.011	-0.012	-0.011	-0.014
GBR	-0.012	-0.012	-0.011	NaN
GEO	-0.016	-0.018	-0.017	-0.015
GHA	-0.010	-0.010	-0.009	-0.008
GIN	-0.013	-0.011	-0.011	-0.014
GMB	-0.016	-0.015	-0.014	-0.002
GNB	-0.008	-0.007	-0.007	-0.009
GNQ	-0.013	-0.012	-0.012	-0.014
GRC	-0.017	-0.017	-0.016	-0.014
GTM	-0.003	-0.003	-0.004	-0.012
GUF	-0.011	-0.012	-0.012	-0.014
GUY	-0.016	-0.016	-0.015	-0.013
HND	-0.004	-0.003	-0.003	-0.009
HRV	-0.019	-0.021	-0.019	NaN
HTI	-0.011	-0.012	-0.012	-0.005
HUN	-0.013	-0.013	-0.012	-0.020
IDN	-0.009	-0.010	-0.010	-0.009
IND	-0.016	-0.016	-0.015	-0.012

Continued on next page

ISO3 Country Code	Model0 Drought Coef. Means	Model1 Drought Coef. Means	Model2 Drought Coef. Means	Model3 Drought Coef. Means
IRL	-0.014	-0.011	-0.012	-0.014
IRN	-0.012	-0.013	-0.012	-0.027
IRQ	-0.015	-0.016	-0.015	-0.031
ISR	-0.011	-0.012	-0.011	-0.020
ITA	-0.012	-0.014	-0.013	-0.015
JAM	-0.017	-0.017	-0.016	-0.010
JOR	-0.008	-0.011	-0.010	-0.023
JPN	-0.010	-0.010	-0.009	-0.014
KAZ	-0.013	-0.013	-0.012	-0.022
KEN	-0.010	-0.010	-0.010	-0.008
KGZ	-0.013	-0.013	-0.013	-0.007
KHM	-0.004	-0.002	-0.002	-0.010
KOR	-0.016	-0.015	-0.014	-0.013
KWT	-0.012	-0.012	-0.012	NaN
LAO	-0.009	-0.010	-0.010	-0.011
LBN	-0.011	-0.012	-0.011	-0.014
LBR	-0.013	-0.014	-0.013	-0.015
LBY	-0.012	-0.012	-0.011	-0.014
LKA	-0.012	-0.010	-0.011	-0.013
LSO	0.001	0.002	0.002	-0.011
LTU	-0.027	-0.029	-0.027	-0.018
LUX	-0.012	-0.012	-0.011	NaN
LVA	-0.013	-0.013	-0.011	-0.014
MAR	-0.014	-0.016	-0.014	-0.017
MDA	-0.024	-0.026	-0.024	-0.037
MDG	-0.013	-0.013	-0.013	-0.010
MEX	-0.010	-0.010	-0.010	-0.011
MKD	-0.020	-0.021	-0.019	NaN
MLI	-0.011	-0.010	-0.009	-0.005
MLT	-0.013	-0.012	-0.011	-0.014
MMR	-0.011	-0.012	-0.012	-0.014
MNG	-0.021	-0.021	-0.020	-0.014
MOZ	-0.013	-0.014	-0.013	-0.012
MRT	-0.006	-0.003	-0.002	-0.017
MWI	-0.020	-0.020	-0.019	-0.013
MYS	-0.013	-0.015	-0.014	-0.013
NAM	-0.030	-0.032	-0.030	-0.059
NER	-0.004	-0.003	-0.003	-0.012
NGA	-0.012	-0.012	-0.012	-0.008
NIC	-0.010	-0.008	-0.008	-0.010

Continued on next page

ISO3 Country Code	Model0 Drought Coef. Means	Model1 Drought Coef. Means	Model2 Drought Coef. Means	Model3 Drought Coef. Means
NLD	-0.014	-0.012	-0.011	-0.014
NOR	-0.012	-0.012	-0.011	-0.014
NPL	-0.011	-0.014	-0.013	-0.013
NZL	-0.008	-0.008	-0.008	-0.011
OMN	-0.011	-0.012	-0.011	-0.014
PAK	-0.014	-0.012	-0.011	-0.005
PAN	-0.011	-0.012	-0.011	-0.013
PER	-0.010	-0.009	-0.009	-0.010
PHL	-0.010	-0.009	-0.009	-0.012
PNG	-0.012	-0.011	-0.011	-0.010
POL	-0.012	-0.012	-0.011	-0.018
PRI	-0.013	-0.014	-0.013	-0.012
PRK	-0.007	-0.006	-0.006	-0.012
PRT	-0.014	-0.016	-0.015	-0.021
PRY	-0.012	-0.012	-0.012	-0.015
PSE	-0.013	-0.012	-0.012	-0.014
QAT	-0.011	-0.012	-0.011	NaN
ROU	-0.015	-0.016	-0.016	-0.012
RUS	-0.012	-0.012	-0.012	-0.013
RWA	-0.011	-0.013	-0.013	-0.011
SAU	-0.012	-0.012	-0.011	-0.014
SDN	-0.010	-0.009	-0.009	-0.012
SEN	-0.038	-0.039	-0.036	-0.017
SLB	-0.013	-0.011	-0.011	-0.012
SLE	-0.012	-0.012	-0.011	-0.014
SLV	-0.016	-0.015	-0.015	-0.015
SOM	-0.008	-0.006	-0.005	-0.014
SUR	-0.011	-0.012	-0.011	-0.014
SVK	-0.012	-0.012	-0.011	NaN
SVN	-0.012	-0.012	-0.011	NaN
SWE	-0.008	-0.012	-0.011	-0.014
SWZ	-0.007	-0.006	-0.006	-0.016
SYR	-0.021	-0.023	-0.021	-0.021
TCD	-0.010	-0.009	-0.008	-0.007
TGO	-0.014	-0.014	-0.013	-0.012
THA	-0.007	-0.006	-0.006	-0.006
TJK	-0.006	-0.006	-0.006	-0.024
TKM	-0.013	-0.012	-0.012	-0.014
TTO	-0.009	-0.010	-0.009	-0.015
TUN	-0.017	-0.019	-0.018	-0.024

Continued on next page

ISO3 Country Code	Model0 Drought Coef. Means	Model1 Drought Coef. Means	Model2 Drought Coef. Means	Model3 Drought Coef. Means
TUR	-0.012	-0.012	-0.011	-0.014
TWN	-0.012	-0.012	-0.012	NaN
TZA	-0.012	-0.013	-0.012	-0.012
UGA	-0.013	-0.014	-0.014	-0.010
UKR	-0.013	-0.011	-0.011	-0.018
URY	-0.014	-0.014	-0.014	-0.017
USA	-0.007	-0.011	-0.011	-0.008
UZB	-0.012	-0.011	-0.011	-0.017
VEN	-0.013	-0.012	-0.012	-0.016
VNM	-0.006	-0.005	-0.006	-0.011
YEM	-0.013	-0.011	-0.011	-0.014
ZAF	-0.013	-0.013	-0.013	-0.007
ZMB	-0.006	-0.004	-0.004	-0.018
ZWE	0.004	0.004	0.003	-0.012
